# Nomenclature for standardized designation of diploid genotypes in genetically modified laboratory animals

**DOI:** 10.1177/00236772231175727

**Published:** 2023-09-06

**Authors:** Peter Dobrowolski, Thorsten Buch, Stefan Nagel-Riedasch

**Affiliations:** 1 GVG Genetic Monitoring GmbH, Leipzig, Germany; 2 Institute of Laboratory Animal Science, University of Zurich, Switzerland; 3 Research Facilities for Experimental Medicine, Charité – Universitätsmedizin Berlin, Germany

**Keywords:** Diagnostic assay, genetics, GM models, organisms and models, PCR, policy, quality assurance/control, techniques

## Abstract

Information about the diploid genotype of a gene-modified or mutant laboratory animal is essential for breeding and experimental planning. It is also required for the exchange of animals between different research groups or for communication with professional genotyping service providers. While there are detailed, standardized rules for creating an allele name of a genome modification or mutation, the notation of the diploid genotype after biopsy and genotyping has not been standardized yet. Therefore, a uniform, generally understandable nomenclature for the diploid genotype of gene-modified laboratory animals is needed. With the here-proposed nomenclature recommendations from the Committee on Genetics and Breeding of Laboratory Animals of the German Society for Laboratory Animal Science (GV-SOLAS), we provide a practical, standardized representation of the genotype of gene-modified animals. It is intended to serve as a compact guide for animal care and scientific personnel in animal research facilities and to simplify data exchange between groups and with external service providers.

## Nomenclature pour la désignation normalisée des génotypes diploïdes des animaux de laboratoire génétiquement modifiés Résumé

L’information sur le génotype diploïde d’un animal de laboratoire mutant ou modifié par un gène est essentielle, non seulement pour la reproduction et la planification expérimentale, mais également pour l’échange d’animaux entre différents groupes de recherche ou pour la communication avec les fournisseurs de services professionnels de génotypage. Bien qu’il existe des règles détaillées et normalisées pour créer le nom d’un allèle de modification ou de mutation génomique, la notation du génotype diploïde après la biopsie et le génotypage n’a pas encore été standardisée. Une nomenclature uniforme, généralement compréhensible, pour le génotype diploïde des animaux de laboratoire modifiés par des gènes s’avère donc nécessaire. Avec les recommandations de nomenclature proposées ici par le Comité de génétique et d’élevage des animaux de laboratoire de la Société allemande de science des animaux de laboratoire (GV-SOLAS), nous fournissons une représentation pratique et standardisée du génotype des animaux génétiquement modifiés. Elle est destinée à servir de guide compact pour le personnel scientifique et de soin des animaux dans les installations de recherche sur les animaux et à simplifier l’échange de données entre les groupes et avec les prestataires de services externes.

## Nomenklatur zur standardisierten Bezeichnung von diploiden Genotypen bei gentechnisch veränderten Labortieren Abstract

Informationen über den diploiden Genotyp eines genveränderten oder mutierten Versuchstiers sind für die Zucht- und Versuchsplanung unerlässlich. Sie werden auch für den Austausch von Tieren zwischen verschiedenen Forschungsgruppen oder für die Kommunikation mit kommerziellen Genotypisierungsanbietern benötigt. Während es für die Erstellung eines Allelnamens einer Genomveränderung oder Mutation detaillierte, standardisierte Regeln gibt, ist die Notation des diploiden Genotyps nach Biopsie und Genotypisierung noch nicht genormt. Daher wird eine einheitliche, allgemein verständliche Nomenklatur für den diploiden Genotyp von genveränderten Versuchstieren benötigt. Mit den hier vorgeschlagenen Nomenklatur-Empfehlungen des Ausschusses für Genetik und Züchtung der Versuchstiere der Gesellschaft für Versuchstierkunde (GV-SOLAS) wird eine praxisnahe, standardisierte Beschreibung des Genotyps genveränderter Tiere vorgelegt. Sie soll als kompakter Leitfaden für das tierpflegerische und wissenschaftliche Personal in Tierforschungseinrichtungen dienen und den Datenaustausch zwischen Gruppen und mit externen Dienstleistern vereinfachen.

## Nomenclatura para la designación normalizada de genotipos diploides en animales de laboratorio modificados genéticamente Resumen

La información sobre el genotipo diploide de un animal de laboratorio modificado genéticamente o mutante es fundamental para la cría y la planificación experimental. También es necesario para el intercambio de animales entre distintos grupos de investigación o para la comunicación con proveedores profesionales de servicios de genotipado. Aunque existen reglas detalladas y estandarizadas para crear un nombre de alelo de una modificación o mutación genómica, la notación del genotipo diploide tras la biopsia y el genotipado aún no se ha estandarizado. Por ello, se necesita una nomenclatura uniforme y generalmente comprensible para el genotipo diploide de los animales de laboratorio modificados genéticamente. Con las recomendaciones de nomenclatura aquí propuestas por el Comité de Genética y Cría de Animales de Laboratorio de la Sociedad Alemana para la Ciencia de Animales de Laboratorio (GV-SOLAS), facilitamos una representación práctica y estandarizada del genotipo de los animales modificados genéticamente. Su objetivo es servir de guía compacta para el personal científico y de cuidado de animales en las instalaciones de investigación animal y simplificar el intercambio de datos entre grupos y con proveedores de servicios externos.

## Introduction

Notation systems for genetically modified and mutant rodents (rats and mice) were initially developed decades ago^1^^,^^2^ and are now updated yearly.^3^ These internationally accepted MGI guidelines (Mouse Genome Informatics, The Jackson Laboratory, Bar Harbor, ME, USA) allow the accurate description and identification of any gene-modified allele and the accompanying background genome. In daily lab and husbandry work, these complex line and strain notations are frequently replaced by shorthand, especially when they are combined with relevant information about the diploid genotype. It is well known that the notation of the diploid genotype differs between and within animal research facilities and commercial genotyping providers and is far from being standardized. We observed this when we performed a random sampling of genotyping results from 11 German research facilities, four of which transferred their genotyping data automatically into their breeding databases (data not shown). This unsatisfactory situation can easily lead to critical errors due to misinterpretation of information. Furthermore, during data exchange, the genotype information needs to be customized for different parties, binding additional resources and introducing more error-prone steps. Examples of such ambiguous notations are the ‘+’ and ‘−’ symbols. Although in classical genetics the symbol ‘+’ was used exclusively to indicate the wild-type and the symbol ‘−’ a mutation (corresponding to enzyme not active),^4^^,5^ nowadays the symbol ‘+’ is often used to indicate the presence of a transgene, while the wild-type allele, on the other hand, is on occasion indicated by ‘−’.^6–8^ Such ambiguous, even contradictory, use of one and the same symbol constitutes a grave problem as it does not allow a correct interpretation of the actual genotype without additional information such as a decoding table. The frequent exchange of gene-modified lines between research groups or animal research facilities means that this ambiguity is a severe source of errors. If notation-related mistakes result in wrong breeding and failed experiments this nomenclature problem is not only a financial but also an animal welfare and scientific problem.

The Committee on Genetics and Breeding of Laboratory Animals of the German Society for Laboratory Animal Science (GV-SOLAS) tasked a subcommittee (the authors of this paper) to develop nomenclature recommendations for the notation of the diploid genotype of genetically modified mice and rats. These recommendations, presented here, are intended as an aid for recording results in routine genotyping of animals during breeding in animal research facilities. They are secondary to the mandatory adherence to the MGI guidelines for the description of mouse and rat lines and strains. Hence, especially when referring to constructs that can be modified by recombinases, a focus was placed on allowing the researcher to have the relevant information in the shortest and most practical way (as a post-typing result), even if this means that the notation may seem incongruent with the MGI nomenclature guidelines. In many instances, the MGI nomenclature will be complemented with a shorthand of the line name, which may support everyday work but does not allow unambiguous identification. To our knowledge, no rules for such shorthands apply and we do not provide any suggestions in that direction.

To achieve the goal of a notation for the diploid genotype, the following criteria were considered in the recommendations presented here:

- Keeping the proven representation of the diploid genotype (e.g. formerly +/−) by use of a ‘/’;
- Use of self-explanatory notation elements wherever possible (e.g. ‘u’ for ‘unknown’ or ‘q’ for ‘questionable’);
- Uniform logic in comparable systems (e.g. Cre or FLP recombinase-mediated modifications);
- Providing the shortest possible notation;
- Avoidance of ambiguity even with complex constructs (e.g. multiple recombinase target sites);
- Openness to novel constructs;
- Compatibility of the syntax with the data import function of spreadsheet and database programs (e.g. avoiding ‘?’ and separator symbols).

Following these criteria, the notation of the genotype documents the allele-specific genetics of the respective individual animal. Again, it does not replace the MGI guidelines, which represent the genetic changes and the background genome present in a respective line, strain or stock. The proposed nomenclature for the annotation of the diploid genotype reflects the result of the biopsy examination. It does not allow any conclusion on germline location, strain, cell type specificity or systemic presence. An overview of all proposed abbreviations is given in Table 1.

**Table 1.** Brief overview of the abbreviations used.

Abbreviation	Genetic modification	Explanation
2	Absence of a second allele	Presence of two different mutations in the one gene locus (e.g. R26 locus), genotyped with two PCRs
del	Deletion	Natural deletion (e.g. according to CRISPR)
dfl	Deleted fl	Allele deleted after recombination of loxP target sequences
dft	Deleted ft	Allele deleted after recombination of frt target sequences
drx	Deleted rx	Allele deleted after recombination of rox target sequences
dvx	Deleted vx	Allele deleted after recombination of vox target sequences
fl	‘floxed’, loxP site	Presence of two or more loxP target sequences
fl66-71	loxP site variant	Example of a combination of loxP-variants in one construct
fl;rx	Allele with loxP and rox sites	Presence of two or more loxP and two or more rox target sequences in one construct
ft	frt site	Presence of two or more frt sites (for FLP recombinase)
ft3	frt site variant	Example of a frt site variant, for example, F3
hu	Humanized allele	DNA sequence replaced by the analogous human sequence
inv; irx	Inversion, inverted	DNA segment is present inversely (flipped); rox sites flanked, inverted DNA segment
ki	Knock-in	Integration of a DNA sequence with known integration site
ko	Knock-out	Function of a gene is abolished
mut	Mutation	Mutation, not further specified
pm	Point mutation (SNP)	Mutation of a nucleotide, single nucleotide polymorphism
q	Questionable finding	No result due to degraded DNA or questionable result (repeat sampling/genotyping recommended)
rx	rox site	Presence of two or more rox sites (for Dre recombinase)
tg	Transgenic	Insertion of a functional DNA sequence (e.g. enzyme activity, such as Cre, FLP, etc.); insertion with known or unknown integration site
u	Unknown integration site	Notation of the second allele in case of unknown integration site (e.g. random insertion transgenes)
vx	vox site	Presence of two or more vox sites (for Vika recombinase)
wt	Wild-type	Notation of unmodified, native allele
xmut/y0, xwt/y0	X chromosome linked mutation	Notation of X- and Y-chromosomal alleles (hemizygous loci in male individuals), the unaffected Y chromosome is designated as y0
x0/ywt, x0/ymut	Y chromosome linked mutation	Notation of X- and Y-chromosomal alleles (hemizygous loci in male individuals), the unaffected X chromosome is designated as x0

## Rules and general principles of genotype documentation

### Rule 1. Unique designation of the wild-type allele

The wild-type allele is always referred to as ‘wt’. In heterozygous genotypes, this is always in second place.

Example 1:

**Table:** 

mut/wt

### Rule 2. The results of genotyping of both homologous chromosomes are given

The sister alleles are separated by ‘/’. Depending on the genotype, these can be two identical (homozygous) or different (heterozygous) alleles. Except for ‘wt’, the genotypes are provided in alphabetical order.

Example 2:

**Table:** 

wt/wt, mut/wt or mut/mut

In the case of genetic modifications on the X or Y chromosome, a second allele is generally missing in male animals. Classical genetics defines such markers as hemizygous. The information of sex chromosome-based inheritance is important for further breeding. To provide clarity here, the corresponding genotypes should be completed with a preceding x or y. In most cases, the mutation is located on only one of the two sex chromosomes. Therefore, ‘x0’ or ‘y0’ is assigned to the chromosome not carrying the gene.

Example 3:

**Table:** 

xwt/y0, xmut/y0 or x0/ymut, x0/ywt	in males if mutation is coupled to only one sex chromosome
xwt/xwt, xmut/xmut or xmut/xwt	in females

### Rule 3. The allele deviating from the wild-type is named precisely and unambiguously

This rule applies provided that the nature of the genetic modification is known.

Example 4:

**Table:** 

del	natural deletion, deletion after CRISPR/Cas9, et cetera
inv	inverted, inversion of a DNA sequence (exception recombinase-mediated, see below)
ki	knock-in, targeted insertion of a DNA sequence
ko	knock-out, targeted deletion of a DNA sequence
mut	mutation, not further specified
pm	point-mutation, SNP
sp	spontaneous mutation, not further specified
tg	transgene, random insertion of a DNA sequence

### Rule 4. Recombinase genes in the genome are annotated with ‘ki’ or ‘tg’

Recombinase genes can be integrated into the genome by targeted mutagenesis or random integration. Thus, the designation ‘ki’ or ‘tg’ are used, respectively. In the latter case the location of the second allele is designated as ‘u’ for ‘unknown’ integration.

Example 5:

**Table:** 

ki/ki, ki/wt or wt/wt	for known integration sites (wt allele determinable)
tg/tg, tg/u (see rule 9) and wt/wt	for unknown integration site

### Rule 5. Recombinase target sites are indicated with two letters (fl, ft, rx and vx) and after recombination by a preceding ‘d’ (deletion) or ‘i’ (inversion). Multiple different sites separated by a ‘;’ in alphabetical order

Constructs with target sequences for recombinases (recombinase target sites) should be labelled with a unique identifier for the specific recombination target sequence. As a rule, the more relevant/precise information should be favoured over a more general one, as, for example, ‘ki’ over ‘mut’. Especially regarding constructs containing recombinase targets, it is not intended to combine, for example, ‘fx’ and ‘ki’ to describe the mechanism and result. With knowledge of the genetic construct and mechanism underlying an intended knock-in event, the more relevant designation is recommended, for example, ‘fl’ over ‘ki’. After a simple homozygous knock-in line has been established by recombination, the relevant information for intercrossing between lines may then be ‘ki’.

Example 6:

**Table:** 

Recombinase	Target sequence	Abbreviation
Cre^9^	loxP	fl
FLP^10^	FRT	ft
Dre^11^	rox	rx
Vika^12^	vox	vx

- Representation of a deleting recombination.

**Figure 1.**
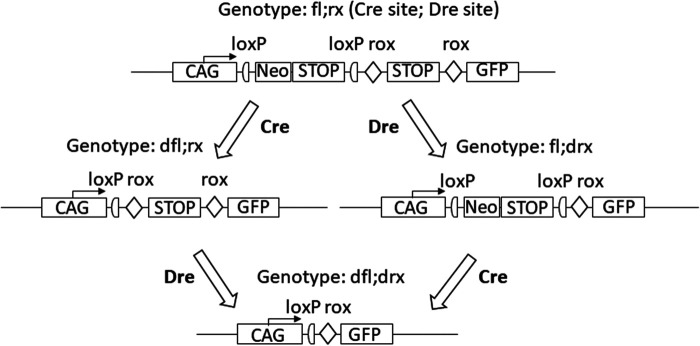
Stepwise, targeted generation of deletion mutants. By selective action of either Cre or Dre recombinase from the initial genotype fl;rx the genotypes dfl;rx, fl;drx and dfl;drx are obtained. CAG - promoter region showing the direction of transcription (here as an example: CAG - promoter), Neo - neomycin resistance gene, GFP - green fluorescent protein without its own promoter, STOP - cassettes with stop codons. In the construct dfl;drx, the transcription of the GFP protein is enabled by the corresponding CAG promoter placed in front of it.

In animals that have both, a ubiquitously expressed recombinase transgene (e.g. deleter-Cre) and a corresponding target allele, the deletion after activation of the recombinase is indicated by a preceding ‘d’ in addition to the recombinase-specific abbreviation (e.g. ‘dfl’) since only the recombination event but no longer the recombinase may be detected in the subsequent generation. In the case of stepwise generation of successive deletions in a mouse line, for example, first with Cre, then with Dre, this representation allows the development of the genotype over the generations to be reliably traced until the target genotype of the mouse line has been generated (see Figure 1).

**Figure 2.**
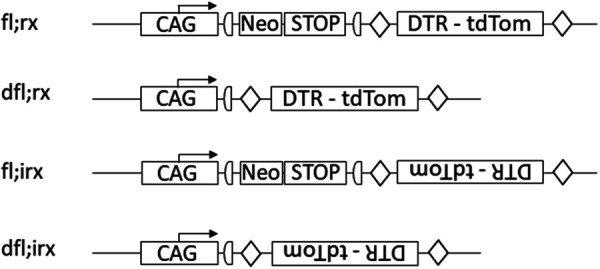
Designation of genotypes for constructs built inversely to the reading frame. CAG - promoter region showing the direction of transcription (here as an example: CAG - promoter), Neo - neomycin resistance gene, STOP - cassettes with stop codons, DTR - tdTom - cassette with diphtheria toxin receptor and fluorescent protein tdTomato without its own promoter. In the construct dfl;rx transcription of DTR-tdTomato is activated by the corresponding CAG promoter placed upstream.

Once the generation of a new mouse line is complete, these animals are designated as a new line using the appropriate MGI nomenclature (e.g. tm1 becomes tm1.1).

Recombinase-mediated deletions are not identical to deletions in the classical sense (by natural base pair loss) or deletions mediated by CRISPR/Cas. In addition to the desired deletion, at least one copy of the respective recombinase binding site (e.g. flox-site) always remains in the genome. Such specific alleles extended by the abbreviation ‘d’ (e.g. dfl or drx) are consequently composed of the deletion and a knock-in of a foreign DNA sequence. The strict distinction alerts the user not to consider a genotype with ‘dfl’ as a simple deletion. The clear assignment of genetic modifications to ‘del’ or ‘dfl’ also simplifies the classification of animals with genomic deletion as ‘natural’ versus genetically modified. In the case of CRISPR/Cas mediated deletion, no foreign sequences remain in the genome, therefore being treated as a ‘natural’ deletion in the context of the genotype and noted as ‘del’.

Example 7:

**Table:** 

fl/fl	becomes dfl/dfl or dfl/fl or genotype
fl/wt	becomes dfl/wt

b. Representation of target site variants.

For simplicity, the abbreviation ‘fl’ symbolizes all genomic situations in which sites can be recombined by Cre. However, for constructs with different loxP-variants, further specifications may be required, for example, fl5171^13^ or fl66-71.^14^ Combinations of loxP-variants in a construct will be separated by ‘-’, for example, fl66-71. The respective deletion or inversion following recombination are again indicated by an additional ‘d’ or ‘i’ at the corresponding position, leading to dfl66-71 or ifl66-71, respectively. This rule also applies to other modifications of recombination sites, for example, FLP target sites (e.g. F3).^15^

Example 8:

**Table:** 

fl66-71/…	becomes dfl66-71 or ifl66-71
ft3/…	becomes dft3

c. Representation of an inverting recombination.

Constructs with a cassette that is inverse to the promoter sequence (flipped) are indicated by a preceding ‘i’; after inversion into the correct reading frame, the ‘i’ is omitted. The same applies to a starting configuration in transcriptional orientation: ‘fl’ becomes ‘ifl’.

Example 9:

**Table:** 

fl;irx/… and fl;rx/…	(see Figure 2)

d. Representation of multiple recombination features and recombination events.

Constructs with sites for more than one recombinase are identified by the respective abbreviation, sorted in alphabetical order and separated by a semicolon. The indication of recombination is performed positionally for the respective enzyme, as given in example 12.

Remaining target sequences or other gene cassettes are not considered when naming the genotypes, such as one rox and one loxP site each as well as the GFP cassette in the genotype ‘dfl;drx’ as in Figure 1.

Example 10:

**Table:** 

fl;ft/…	Construct with sites for Cre and FLP
fl;rx/…	Construct with sites for Cre and Dre (see Figure 1)

Example 11:

**Table:** 

fl;ft;rx;vx/…	Genotype of a reporter mouse line with sites for the Cre, Dre, FLP and Vika recombinases^16^

Example 12:

**Table:** 

fl;rx/…	becomes either dfl;rx or fl;drx (see Figure 1)
Genotypes:	dfl;rx/dfl;rx or dfl;rx/wt or dfl;rx/fl;drx or fl;drx/wt etc.

### Rule 6. Each genetically modified region of a mouse line is recorded only once in the database

**Figure 3.**
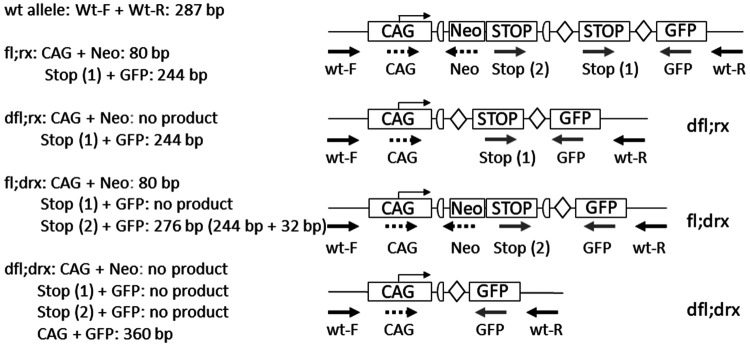
Combination of different genotyping results into one final record of alleles presented in Figure 1. Derivation of the final genotype of a complex construct from Figure 1 based on the results of multiple independent polymerase chain reaction (PCR) analyses. The length of a loxP site in this figure is 34 bp. Therefore, the PCR product for stop-GFP is larger by this value for the fl;drx genotype than for dfl;rx or fl;rx. The arrows each represent a PCR primer in the corresponding orientation (forward, reverse).

The single-entry rule also applies to complex genetic constructs where the results of several independent PCR reactions must be combined into one result (see Figure 3, as well as Supplementary material Figure 1 online, annotating the final allele ‘fl;rx’ instead of separate presentation of genotyping results of the neomycin resistance gene (Neo) and green fluorescent protein (GFP)). This facilitates correct selection of suitable animals for subsequent matings without the need for additional background information.

As shown in Figure 3, the results of the genotyping are presented in compressed form. They result logically from the respective matings of the parents.

Example 13:

**Table:** 

fl;rx/wt, or dfl;rx/dfl;rx et cetera.	

### Rule 7. The combination of two genetically unrelated, gene-modified alleles located at the same locus leads to two database entries, with a ‘2’ for the second allele

**Figure 4.**
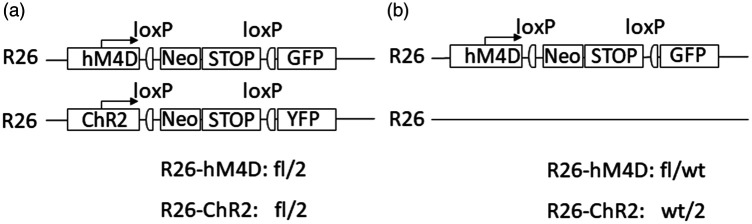
Genotype options of parents with different, genetically unrelated constructs in the identical corresponding allele position. Mating of parents with two different, genetically unrelated constructs located at identical positions in the genome (e.g. in the R26 locus) results in offspring which may possess both genetic constructs simultaneously (and no wild-type allele). The genotypes are then referred to as ‘fl/2’ for both markers and recorded independently (a). Depending on the mating scheme, in the case of positive detection of the wild-type allele, the genotype is either ‘fl/wt’ or ‘wt/2’ (b).

For certain experimental setups two different modifications have been introduced into the same locus (e.g. R26-hM4D (fl/fl) and R26-ChR2 (fl/fl); Figure 4) and crossed together to obtain heterozygous animals for both modified alleles. Examples of such a situation are the various insertions into the Gt(ROSA)26Sor locus (commonly known as Rosa26 or R26 locus).^17^ In contrast to the representation of the two alleles of a gene with multiple possible recombination outcomes (yet still all located within the same construct) as described in rule 6, the genotypes of two independent mutations present in the same locus but on sister chromosomes need to be recorded separately. Since the second, homologous, allele is occupied by the allele of the other, different mutation, the position of the second allele is marked with ‘2’. The ‘2’ here indicates the presence of a second, unrelated, construct at the identical location on the sister chromosome. Exceptions are genotypes with a wild-type allele or other alleles unambiguously distinguishable from each other (e.g. fl/wt) if these can be clearly derived from the respective mating scheme (see example 15, and Figure 4(b)).

Example 14: 

**Table:** 

R26-hM4D (fl/fl) ×R26-ChR2 (fl/fl)	(see Figure 4(a)))
Genotypes of the offspring:	R26-hM4D: fl/2
	R26-ChR2: fl/2

Example 15:

**Table:** 

R26-hM4D (fl/wt) ×R26-ChR2 (fl/fl)	(see Figure 4(b))
Genotypes of the offspring:	R26-hM4D: fl/2 or wt/2
	R26-ChR2: fl/2 or fl/wt

### Rule 8. Use of the character ‘q’ to indicate questionable, ambiguous findings

Occasionally, due to technical problems, the genotype cannot be determined beyond doubt. This is the case if the DNA sample is already highly degraded and larger PCR products are no longer amplified, but short PCR products are still detectable. Should the wt and mut allele be represented as one longer and one short DNA fragment, the failure of the larger product may lead to misinterpretation with respect to genotype. Suboptimal PCR conditions may also lead to preferential amplification of the short PCR product. For such situations, the abbreviation ‘q’ is used for the allele that cannot be identified, indicating that re-genotyping is suggested. Instead of a ‘?’, a ‘q’ for ‘question mark’ is used here to ensure database compatibility.

Example 16:

**Table:** 

q/wt or mut/q or q/q	

### Rule 9. Unknown integration site of a genetic modification is annotated with a ‘u’ for the second allele

If the integration site of a genetic modification is not known, in contrast to known integration sites, no specific primers can be generated with which the wild-type allele can be detected. Breeding of such animals may lead to offspring carrying the transgene on one or both alleles. However, this cannot be determined by conventional PCR.

In the case of proof of the presence of genetic modification, no statement can be made about the second allele. The value ‘u’ for ‘unknown’ is assigned for this situation. This means that either the wild-type allele or a second allele of the corresponding genetic modification is present.

Although classical genetics defines exclusively the X- and Y-chromosomal markers described under rule 2 as hemizygous, constructs with unknown integration sites that can be classified genetically in a completely different way are also regularly described as hemizygous.^18^

Example 17:

**Table:** 

tg/u × tg/u progeny with resulting genotype wt/wt and tg/u, but only in exceptional cases tg/tg, if homozygosity for the transgene can be proven based on the breeding scheme or alternative, molecular biological methods.

### Rule 10. Annotation of genotyping results must allow for automated exchange with third parties and transfer into animal husbandry databases

The automated transfer of genotyping results from an external finding is done either via a csv or a txt file (see also Supplementary Figure 2). Beforehand, the respective possible allele combinations (e.g. wt/wt, fl/wt, etc.) must be released separately in the database for each mutation. Since the name of the mouse line is irrelevant for the data import, only the exact sample ID and the mutation name are required for the successful assignment of the results.

The following requirements must be met in the livestock database:

- All permissible genotypes for the respective mutation must be defined and released separately for each mutation;
- The mutation name must not contain special characters, such as ‘?’ (question mark), ‘_’ (underscore), ‘/’ (slash) or ‘,’ (comma), as these lead to problems in assigning the analysis results or in computer-aided processing.

The following information must be available at the analytical laboratory:

- Sample ID, identical to the sample name in the database;
- Spelling of the mutation name, identical to the mutation name in the database, including spaces, upper and lower case;
- Permissible genotypes for the respective mutation stored in the database.

## Discussion

The MGI guidelines provide a standardized way of naming and identifying different mouse lines, stocks and strains.^1–3^ The latest large addition to this rule set was the result of the endonuclease revolution,^19^ starting with Zinc finger and Tal-effector nucleases and eventually leading to the CRISPR/Cas technology. Having a compatible nomenclature system for naming mutant lines (for review: Sundberg and Schofield^20^) and, as proposed here, for the diploid genotype of the individual animal has several benefits for everyday lab work. The consistent designation of animal-related data among researchers helps to prevent confusion and errors when communicating about or referring to specific mouse lines and breeding results. With the nomenclature system recommended here, researchers can easily identify the specific diploid genotype of individuals of a specific mouse line. This information is critical for understanding the phenotype and behaviour of each line, as well as for comparing results between different studies. It also supports the appropriate breeding of the animals, applying Mendelian rules.^21^ A standardized genotype notation system makes it easier to track the history and origin of each individual mouse, which is vital for ensuring that mouse lines are properly maintained, stored and shared between different research groups.^22^ For instance, we know of designations for diploid genotypes having been changed over time, making historical analyses of pedigrees unnecessarily complex or even impossible. A standard notation system further streamlines the necessary documentation and data-sharing when animals are exchanged between labs and institutions or when external genotyping services are used. This helps to promote collaboration and speed up the pace of scientific discovery. Furthermore, the proposed genotype notation system makes it easier to electronically manage and organize data related to genetically modified animals. Such management systems help to reduce errors and improve the accuracy of data analysis and overcome paper-related error-prone data exchange throughout the whole scientific data pipeline from the decision for an animal experiment to consistent storage of scientific data and animal-derived specimen in tissue repositories.

Our recommendations do not intend to replace the MGI guidelines for allele, stock and strain designation but serve as an addition to make genotype recording, in most cases after genotyping by means of molecular biology methods, consistent over labs and institutions. We do not address the fact that in many research animal facilities abbreviations for line names are common. While these are used together with a genotype notation, we do not see an easy consensus on how such common names and abbreviations should be generated and how to deal with established abbreviations and common names, especially since many of these names are easy to remember and hence support communication about lines, for example, Tiger, Confetti.^23^^,^^24^

Being a source of concern for a long time, a standardized nomenclature for the notation of the diploid genotype has surprisingly not been considered so far. Therefore, we also aim at a harmonization of genotype notation enabling the community of researchers generating and using gene-modified and mutant rodents to establish in their respective institutions a clear record of present and past animal genotypes. We also believe that further discussion is necessary about this particular aspect of nomenclature. This discussion could also take place within the established International Committee for Standardized Genetic Nomenclature in Mice.

Overall, we are confident that the rules for the standardization of notation of the diploid genotype, as presented here, are compatible with everyday lab work and will help to improve the efficiency, accuracy and transparency of research involving genetically modified mouse lines. Finally, it will help to adhere to the 3R principles in terms of reduction and refinement.

## Data Availability

The data underlying examples and figures are available from the corresponding author upon reasonable request.
